# Human Oral-Associated *Capnocytophaga* Species and Their Clinical Relevance

**DOI:** 10.3390/microorganisms14071512

**Published:** 2026-07-11

**Authors:** Eija Könönen, Mervi Gürsoy, Ahmed Algahawi, Rory Munro Watt

**Affiliations:** 1 Institute of Dentistry, University of Turku, Lemminkäisenkatu 2, 20520 Turku, Finland; mekrgu@utu.fi (M.G.); aaelga@utu.fi (A.A.); 2 Faculty of Dentistry, The University of Hong Kong, Hong Kong, China; rmwatt@hku.hk

**Keywords:** *Capnocytophaga*, biofilms, classification, dysbiosis, gram-negative bacteria, infections, oral cavity

## Abstract

Members of the *Capnocytophaga* genus are common residents of the oral cavity of humans and some animals, notably dogs and cats. In humans, *Capnocytophaga* species have received considerably less attention than their canine compatriots due to their more nuanced roles in health and disease. Whilst they are commonly associated with oral health, many aspects regarding their respective functions, inter-species interactions, and activities within oral microbial biofilm communities remain to be established. Furthermore, there are increasing numbers of reports linking certain human oral-associated *Capnocytophaga* species with systemic infections, suggesting their activities as low-level pathogens. However, clinical data are currently scanty. In this narrative review, we give an overview of human oral-associated *Capnocytophaga*, including their taxonomy and genomic compositions, challenges in their species-level identification, their colonization patterns within the oral cavity, potential pathogenic features, and their clinical relevance in oral and non-oral infections. We discuss the similarities and differences between key aspects of human and animal oral-associated species of *Capnocytophaga*, including experimental methods used for their comparative functional analysis.

## 1. Genus *Capnocytophaga*

Members of the *Capnocytophaga* genus are known as common residents of the oral cavity of humans and some animals. In this review, our objective is to give an overview of human oral-associated *Capnocytophaga*, focusing on their current taxonomy and genomic compositions, challenges in their species-level identification, their colonization patterns in children and adults, as well as their clinical relevance in oral and non-oral infections.

### 1.1. Taxonomic Considerations

**Figure 1:**
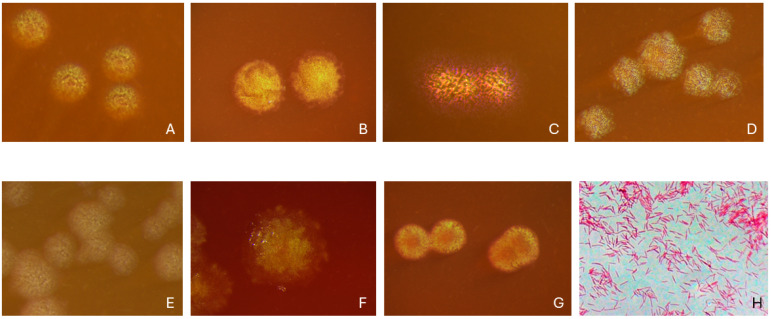
Colonial Morphology of Human Oral *Capnocytophaga* Strains Grown on Brucella Agar for Three Days: *Capnocytophaga* genospecies AHN8471 (**A**), *C. gingivalis* CCUG 9715^T^ (**B**), *C. granulosa* CCUG 32991^T^ (**C**), *C. haemolytica* CCUG 32990^T^ (**D**), *C. leadbetteri* AHN8855^T^ (**E**), *C. ochracea* AHN37380 (**F**), and *C. sputigena* CCUG 9714^T^ (**G**), and Gram Stain of *C. haemolytica*, a Gram-Negative Rod (**H**) (own laboratory data).

In the late 1970s, strains formerly classified as *Bacteroides ochraceus* and Centers for Disease Control (CDC) group DF-1 (Dysgonic fermenter 1) were re-classified into the novel genus *Capnocytophaga* with three novel species, including *C. gingivalis*, *C. ochracea*, and *C. sputigena* [1,2,3], and the novel genus name was validated in 1982. Since then, the genus has expanded, with the species being phenotypically similar; they grow under anaerobic to microaerophilic conditions, preferring an atmosphere enriched with CO_2_ (i.e., capnophilic), producing yellow–orange or beige, speckled colonies that typically glide on the agar surface, and the cells are thin, slender, elongated rods with a negative Gram stain reaction (Figure 1) [1,4]. The identification of human oral-associated *Capnocytophaga* at the genus level is feasible based on the above-mentioned basic phenotypic characteristics; however, conventional biochemical testing is not reliable for identifying the members of the genus to the species level, which requires more advanced techniques.

**Figure 2:**
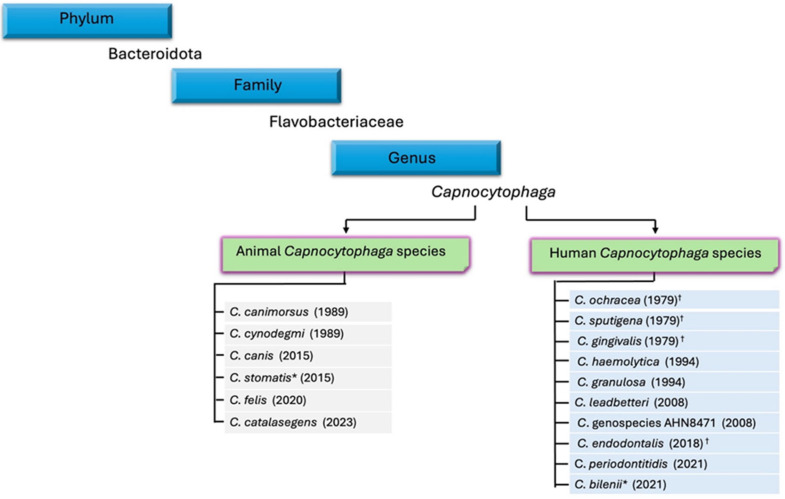
Taxonomy and timeline of currently recognized human- and animal-associated *Capnocytophaga* species. ^†^ The year in parentheses indicates the first description of each species; *C. ochracea*, *C. sputigena*, and *C. gingivalis* were validly published in the Approved Lists of Bacterial Names in 1982, and *C. endodontalis* was validly published in 2025. * Taxa that have not yet been officially validated.

The genus *Capnocytophaga* currently contains eight human and five animal species that are validly published, while two ‘species candidates’ are still awaiting validation (Figure 2). The genus *Capnocytophaga* belongs to the family Flavobacteriaceae within the phylum Bacteroidota. *C. ochracea* is the type species of the genus.

While *Capnocytophaga* species of animal origin are well known for their role as important pathogens, especially *C. canimorsus* in bite infections [5,6], data on the role of human oral-associated *Capnocytophaga* species are rather fragmentary in the literature. However, all eight validly published *Capnocytophaga* species in humans are reported in the List of Recommended Names for bacteria of medical importance (LoRNs) because of the recorded risk group of the species themselves [7], and all human oral-associated *Capnocytophaga* species validly published before 2020 are regarded as established pathogens infecting humans [8].

### 1.2. Milestones in Capnocytophaga Genome Sequencing

The type strain of *C. ochracea* (VPI 2845^T^ = DSM 7271^T^ = ATCC 33596^T^) was the first *Capnocytophaga* strain to be genome sequenced in 2009 (GenBank accession: NC_013162) [9]. This revealed a single chromosome of 2,612,955 bp, encoding 2252 genes, with a G + C content of 39.6%: genomic features that are typical of *Capnocytophaga* taxa of both human and animal origin. The genome sequence of the ATCC 33624 type strain of *C. gingivalis* was also deposited in 2009 (GCA_000174755.1), but this was not accompanied by a publication.

Subsequent milestones included the elucidation of the first animal oral-associated *Capnocytophaga* genome sequence, that of *C. canimorsus* strain Cc5 [10], and the publication of 12 complete *Capnocytophaga* reference genomes by a team from the US CDC, including strains of *C. sputigena*, *C. leadbetteri*, *C. gingivalis* (all of human origin); and *C. canimorsus*, *C. cynodegmi*, and *C. canis* (including the CcD38 type strain), and *C. stomatis* (all of canine origin) [11].

The formal descriptions of the following human *Capnocytophaga* species were accompanied by genome sequence data for the type strains: *C. endodontalis* ChDC OS43^T^ (KCTC 5562^T^ = JCM 32133^T^) originally isolated from a human refractory periapical abscess (Korea) [12]; *C. periodontitidis* p1a2^T^ (CGMCC 1.17337^T^ = JCM 34126^T^) isolated from subgingival plaque from a deep periodontal pocket (Beijing, China) [13]. ‘*C. bilenii*’ Marseille-Q4570^T^ (CSUR Q4570^T^) (not yet validly published) was isolated from dental plaque of a 25-year-old male with gingivitis (Marseille, France) [14].

The taxonomic standing of *C. endodontalis* remains controversial, and it has previously been noted that *C. periodontitidis* and *Capnocytophaga* genospecies (genomospecies) AHN8471 [15] may represent strains belonging to the same (or overlapping) species [16]. Data from our ongoing genomic analysis of human oral *Capnocytophaga* isolates suggest that strains originally assigned to *Capnocytophaga* genospecies AHN8471 (namely strains AHN8471, AHN8751, AHN9528, AHN9576, and AHN9798) encompass both the *C. endodontalis* and *C. periodontitidis* species (unpublished data). Hopefully, this taxonomic issue will be resolved in the near future through detailed comparative genomic analysis of additional strains, combined with results from a comprehensive polyphasic characterization.

In chronological order, the descriptions of the following animal *Capnocytophaga* species were accompanied by genome sequence data for the type strains: *C. cynodegmi* ATCC 49044^T^ (CIP 103937^T^ = DSM 19736^T^; from the dog oral cavity) [17]; ‘*C. stomatis*’ W5 (from the dog oral cavity; not yet validly published) [18]; *C. canis* (the first publication described three strains from this species: HP20001, HP33001, and HP40001; isolated from the dog oral cavity but not the type strain) [19]; *C. felis* (KC07070^T^ = JCM 32681^T^= DSM 107251^T^, isolated from the cat oral cavity) [20]; *C. catalasegens* (KC07084^T^ = JCM 32682^T^ = DSM 107252^T^, isolated from the cat oral cavity) [21].

Human and animal strains of *Capnocytophaga* contain a single chromosome and have genome sizes ranging from ca. 2.4–3.4 Mb, with G + C contents ranging from ca. 33.5 to 44%. Strains encode ca. 2150–3150 genes. Currently, the genome of *C. endodontalis* ChDC OS43 is the largest (GCA_002209445.1; ca. 3.41 Mb, 3132 genes) [12], and *C. canimorsus* 7120 is the smallest (GCA_002302565.1; ca. 2.41 Mb, 2136 genes) [11]. Human and animal *Capnocytophaga* genomes generally contain three or four identical sets of the 5S, 16S, and 23S rRNA genes.

### 1.3. Taxonomy Beyond ‘Species’ Boundaries

The expanded Human Oral Microbiome Database (eHOMD) has been a ‘game-changing’ resource for researchers investigating human oral microbial ecology and the etiology of oral diseases [22,23]. By defining ‘species-level’ phylotypes named ‘human microbial taxon’ (HMT), formerly called ‘human oral taxon’ (HOT), within a hierarchical taxonomic and phylogenomic framework, the eHOMD enables researchers to use DNA sequence-based approaches to identify and classify bacterial taxa originating from human oral (and nasopharyngeal) niches. Focusing on *Capnocytophaga*, it should be noted that the animal species *C. canimorsus*, *C. canis*, *C. cynodegmi*, *C. stomatis*, *C. felis*, and *C. catalasegens* are not included in the eHOMD, even though they may be detected in serious infections originating from animal bites or wound-licking infections [5,6]. Many of these companion animal-origin taxa have been categorized within the canine oral microbiome [24] and/or feline oral microbiome [25] taxonomic frameworks.

**Figure 3:**
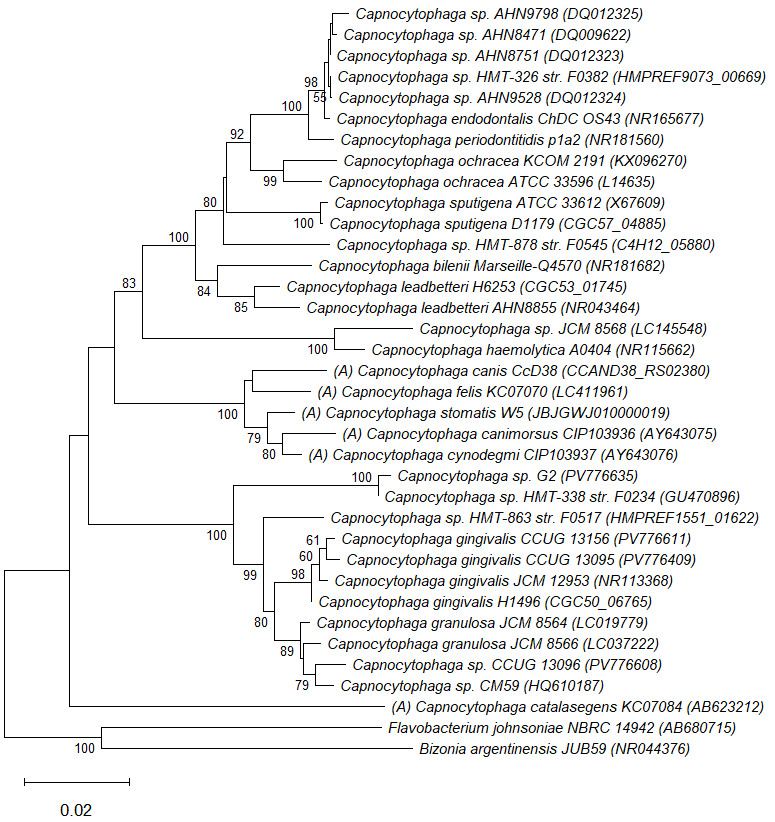
Neighbor-joining (NJ) phylogenetic tree of 16S rRNA gene sequences from representative *Capnocytophaga* strains. The respective strain names are shown to the left of the corresponding branch tips. (A) Preceding the strain name indicates that the strain is of animal origin. The accession codes or gene loci of the DNA sequences used to construct the NJ tree are shown in parentheses. Sequences were aligned using BioEdit version 7.2.0. The NJ tree was constructed using MEGA v12 [28], and bootstrap support values (above 50) from 1000 bootstrap replicates are shown at the respective branch nodes. HMT = human microbial taxon reference numbers according to the expanded Human Oral Microbiome Database (eHOMD) v4.2Φ [23]; str. = strain; sp. = species. 16S rRNA sequences from *Flavobacterium johnsoniae* and *Bizonia argentinensis* (both in the family Flavobacteriaceae) are included as outgroups.

A neighbor-joining (NJ) phylogenetic tree of 16S ribosomal RNA (rRNA) sequences from representative *Capnocytophaga* taxa of human and animal origin is shown in Figure 3. This includes selected taxa from the HOMD (HMT-338, HMT-863, HMT-878, and HMT-326), as well as several strains that we have recently genome-sequenced, with their genomic sequence data deposited in NCBI GenBank. This includes *Capnocytophaga* sp. G2 (GCA_035282625.1, which corresponds to HMT-338), *C. gingivalis* (HMT-337) strains CCUG 13156 (GCA_035282365.1) and CCUG 13095 (GCA_035282545.1), and another CCUG reference strain, which is listed as ‘*C. gingivalis*’ CCUG 13096 (GCA_035282585.1), but which corresponds to HMT-471 [26], along with the genome-sequenced strain *Capnocytophaga* sp. CM59 (GCA_000293175.1) [27]. Both *Capnocytophaga* sp. CCUG 13096 and sp. CM59 are most closely related to *C. granulosa* (HMT-325). It should be noted that the eHOMD is a dynamic and evolving database, and there are numerous publications, as well as genome-sequenced reference strains, that classify *Capnocytophaga* taxa as HMT (e.g., *Capnocytophaga* sp. oral taxon 903 strain W10654) that are not listed in the current version of the eHOMD (v4.2φ). Somewhat confusingly, *C. periodontitidis*, *C. endodontalis*, and *Capnocytophaga* genospecies AHN8471 all appear to correspond to HMT-326, which is defined as being *C. periodontitidis* (eHOMD v4.2φ).

## 2. Colonization and Presence as Residents of the Oral Microbiota at Different Ages and Locations of the Mouth

The primary colonization site of *Capnocytophaga* is the oral cavity. In a longitudinal study on the establishment of oral anaerobes in the saliva of 44 infants during the first year of life, *Capnocytophaga* was categorized as a late colonizer, the detection frequency being 0% at the age of two months, 5% at the age of six months, and then increasing to 20% at the age of 12 months [29]. Earlier, another set of longitudinal data on the occurrence of gram-negative anaerobes in the oral cavity of 21 children demonstrated significant increases in the occurrence of *Capnocytophaga* species from 20% at the predentate stage to 100% at the dentate period (age range 24–41 months) due to the eruption of teeth and formation of gingival crevices with new oral surfaces to be colonized [30]. This culture-based observation was consistent with a recent systematic review where a total of 34 studies using 16S ribosomal RNA (rRNA) gene-targeted sequencing were included to provide comprehensive information on the oral microbiome at birth, early childhood, and adolescence [31]. It was shown that *Capnocytophaga* is among the genera that become dominant with a high level of abundance during the second year of life. In other words, human *Capnocytophaga* species are residents of the oral microbiota from early life onward, their preferred sites being dental biofilms, but they are also present on other oral surfaces. In a culture-based study of suspected periodontal pathogens in 67 children aged five to seven years, 67% of subgingival plaque, 27% of tongue surface, 36% of saliva, and 56% of tonsil samples were positive for *Capnocytophaga* [32].

Due to conflicting data on different *Capnocytophaga* species colonizing young children, 102 isolates from 12 children were selected from a culture-based study of 23 Finnish children aged two to three years [30] and were characterized using cluster analysis of multilocus enzyme electrophoresis profiles and partial 16S rRNA gene sequences by Frandsen et al. [15] to identify the genus-level isolates to the species level. Another focus was on the diversity of *Capnocytophaga* within children, and despite the limited number of isolates available from each child, intra-subject diversity was observed. The phylogenetic and phenotypic analyses led to the description of a novel *Capnocytophaga* species, *C. leadbetteri*, and a novel *Capnocytophaga* genospecies AHN8471 (=genomospecies AHN8471). Among the 102 isolates, the highest prevalence rates were observed for *C. ochracea* and *C. sputigena*, and also *C. granulosa*, *C. leadbetteri*, and the novel genospecies were present at low frequencies, whereas *C. gingivalis* and *C. haemolytica* were not detected [15].

Three PCR-based studies [33,34,35] examined the presence of *Capnocytophaga* at the species level in Japanese children. In dental plaque samples collected from children aged 2–12 years, *C. ochracea* and *C. gingivalis* were most prevalent, being found in 100% and 96% of the 25 healthy children and in 89% and 85% of the 85 children with gingivitis, respectively, while corresponding prevalence rates for *C. sputigena* were 48% and 37%, respectively [33]. In another study, the prevalence rates in subgingival plaque of 78 children aged three to nine years were 36% for *C. ochracea*, 50% for *C. gingivalis*, and 72% for *C. sputigena* [35]. Their findings contrasted with the absence of *C. gingivalis* observed in Finnish children aged 2–3 years [15]. The discrepancy may be explained by differing identification criteria and/or ethnic and/or geographical differences between the studies. Ooshima et al. [34] performed a two-year longitudinal study, where plaque and saliva samples from 119 periodontally healthy children aged 2–15 years were examined using a PCR-based approach, with *C. ochracea* and *C. sputigena* included among the 10 species targeted for study. In saliva, *C. ochracea* and *C. sputigena* were very frequently detected at all ages, but, surprisingly, were less frequent in plaque samples.

The mixed dentition stage, i.e., the period from 6 to 12 years of age, is characterized by the existence of both primary and secondary teeth. This is considered a critical transitional period for pediatric dental patients and was the focus of a recent 24-month randomized controlled trial by Zeng et al. [36] as regards the impact of sodium fluoride varnish alone or combined with oral health education, or oral health education alone, on caries increment and dental plaque microbiota. Significant changes were observed in the genus *Capnocytophaga* at both the 6- and 12-month assessments. After the cessation of interventions, the 24-month findings revealed that the distribution of *Capnocytophaga* became similar among the three intervention groups. *Capnocytophaga* was among the genera showing dynamic shifts in supragingival plaque composition. At the 12-month intervention, *C. granulosa* was less abundant in children who did not receive fluoride varnish, but the underlying reasons remain obscure [36]. A study comparing subgingival plaque findings between young African–American individuals (aged 5–21 years) with localized aggressive periodontitis (i.e., rapidly progressing periodontitis with an incisor-molar distribution) and their healthy siblings and healthy controls reported that *C. granulosa* was an abundant species in healthy individuals [37]. Subgingival samples from 73 generally healthy Bulgarian children, aged 10–14 years, were analyzed for the presence of nine target species, including *C. gingivalis*, by real-time PCR [38]. Unlike other periodontal species, *C. gingivalis* proved to ubiquitously colonize both periodontally healthy children and children with gingival inflammation, and was found in high quantities regardless of clinical status. One Brazilian study used PCR to determine the presence of 11 target species in gingival crevicular fluid samples collected from the first permanent molars and second deciduous molars, and two upper and two lower permanent incisors of diabetic and non-diabetic children aged 7–13 years [39]. Only *C. sputigena* and *C. ochracea* were associated with gingivitis in children with type 1 diabetes. Compared with non-diabetic children, diabetic children had significantly higher levels of *C. sputigena* for all tooth types and *C. ochracea* for permanent teeth.

In a PCR-based study [40], plaque samples were collected from 300 Indian individuals, including 100 periodontally healthy individuals, 100 with gingivitis, and 100 with periodontitis; of these, 87%, 77%, and 73%, respectively, were positive for *Capnocytophaga*. In the healthy group, *Capnocytophaga* detection was most common in young (aged 18–29 years) individuals. Prevalence rates of *Capnocytophaga* species varied as follows: *C. ochracea* 36.3%, *C. granulosa* 32.7%, and *C. gingivalis* 10%, whereas *C. sputigena*, *C. haemolytica*, *C. leadbetteri*, and *Capnocytophaga* genospecies AHN8471 (*C. periodontitidis*/*C. endodontalis*) were not found. The absence of these species, especially *C. sputigena*, was speculated to be due to ethnic and/or geographical differences, but also methodological aspects [41] could partly explain the wide variation between studies.

Socransky’s microbial complex theory [42] includes three *Capnocytophaga* species, *C. gingivalis*, *C. ochracea*, and *C. sputigena*, which are placed within the green complex. “Large plaques” on teeth have been shown to exhibit increased proportions of green and orange complex species [42]. In the green complex, both *C. ochracea* and *C. gingivalis* appeared at high levels on teeth with large amounts of supragingival plaque, which was collected from adult individuals [43,44]. In a study by Teles et al. [45], the focus was on revealing the ecological order of species succession during supra- and subgingival biofilm redevelopment after careful professional dental cleaning, followed by seven days of no oral hygiene. In supragingival plaque from periodontally healthy individuals, the mean proportions of *C. gingivalis* began to increase significantly at two days and continued to increase through four and seven days, while the proportions of *C. ochracea* and *C. sputigena* increased significantly at four days. In subgingival plaque from healthy individuals, significant increases were observed in the mean proportions of *C. gingivalis* at four days and at seven days of *C. sputigena* [45].

Li et al. [46] examined supragingival plaque samples collected before and after scaling at several time points from 40 individuals divided into three periodontal states, representing periodontal health, gingivitis, or periodontitis, with the aim of identifying similarities and differences in plaque remodeling processes. *Capnocytophaga* was among the few genera that had a significant correlation with the most clinical indicators, including bleeding index, plaque index, and percentage of bleeding sites on probing at different time points in the three periodontal states [46]. In a recent longitudinal one-year study, using full-length 16S rRNA and metagenomic sequencing, Zhou et al. [47] examined subgingival plaque samples collected from 30 periodontally healthy Chinese individuals, aged 18−30 years, following ultrasonic scaling (considered a perturbation to the subgingival microbiota) at 12 sequential time points. Microbial dynamics at the species and functional levels were characterized. According to their analysis, the subgingival microbiota reconstruction was mediated through coordinated changes in ecologically linked species groups, and significant positive correlations were observed among member taxa within each functional module. Within one of the modules, *C. gingivalis*, *C. granulosa*, *C. sputigena*, and *C. leadbetteri* were among the high-abundance species, with the novel species *C. endodontalis* (*Capnocytophaga* genospecies AHN8471/*C. periodontitidis*) also identified. Interestingly, within this late-stage module of the reconstructive process, enriched with specific functional capacities, bacterial taxa showed the highest pathogenic potential [47].

In traditional Chinese medicine, the appearance of tongue coatings has been used by clinical practitioners to differentiate ‘Cold’ and ‘Hot’ syndromes, which are considered to play roles in gastrointestinal diseases. Jiang et al. [48] investigated the microbiome composition of tongue coating samples from 19 middle-aged patients with chronic atrophic gastritis (nine diagnosed with Cold Syndrome and 10 with Hot Syndrome) and from eight young adults with no stomach discomfort, using Illumina 16S rRNA amplicon sequencing. Typical tongue coatings in Cold Syndrome patients were ‘white and greasy’, and those in Hot Syndrome patients were ‘yellow and dense’, with their respective tongue-coating microbiomes differing significantly. *Capnocytophaga*, *C. sputigena*, and *C. haemolytica* were detected in ‘Hot’ tongue microbiota-imbalanced networks of genus-classifiable operational taxonomic units (OTUs). It was concluded that the appearance of the tongue coating reflects the imbalanced tongue-coating microbiome in the entire human ecosystem [48].

In a more recent study, Chen et al. [49] collected swab samples from the tongue dorsum of 94 healthy Chinese individuals, which represented eight different tongue coating types with regard to color, thickness, and moisture appearance, for analysis by metagenomic sequencing, to investigate their respective microbial compositions. *C. sputigena* was found within the ‘yellow and thin’ tongue coating, positively correlating with *Neisseria elongata*. Furthermore, due to potential differences in the same tongue coating between individuals with and without disease, a comparison was performed between healthy individuals with a ‘yellow and thick and greasy’ coating and an additional, corresponding diabetic group, showing clear distinctions concerning the high abundance of *Capnocytophaga* in diabetic patients [49].

The impact of oral hygiene and periodontal inflammation on the prevalence of *C. ochracea* and *C. sputigena* on the tongue surface of 136 older Japanese individuals (age range 36–91 years) was investigated using real-time PCR [50]. The prevalence rates for *C. sputigena* and *C. ochracea* were 83% and 68%, respectively. It was observed that oral hygiene was poor in individuals positive for *Capnocytophaga* compared with those without *Capnocytophaga*, and the detection of both species was associated with mild periodontal inflammation.

Evidence from current experimental gingivitis studies where dental hygiene is discontinued, thus allowing natural bacterial accumulation, demonstrates a significant degree of variation in human inflammatory response to dental biofilm formation [51,52]. Among 40 periodontally and systemically healthy young adults, slow responders had a high abundance of *Streptococcus* and a low abundance of *Capnocytophaga* in supragingival plaque at baseline, whereas rapid responders had significantly higher abundances of *Capnocytophaga* at baseline compared with moderate and slow responders [52]. During the 14-day experimental gingivitis period, slow and moderate responders had significantly higher abundances of *Capnocytophaga* than rapid responders, with *C. sputigena*, *C. granulosa*, and *C. leadbetteri* being among the predominant bacterial species. Slow responders showed microbial resilience towards gingival inflammation, indicating their potential to return to baseline microbial conditions [52].

In a culture-based study on gram-negative anaerobic species in saliva and subgingival samples from 30 women (mean age 30 years), the majority harbored *Capnocytophaga* organisms, and when positive subgingivally, 64% of the saliva samples were simultaneously positive [53]. Manzoor et al. [54] used a metagenomic approach to analyze 120 saliva samples from young adults, divided into two age- and sex-matched groups, one half diagnosed as periodontally healthy/localized gingivitis (controls) and the other half as generalized gingivitis/initial periodontitis (cases). The latter group may represent a transition phase between a still reversible inflammation of the gingiva and early-stage non-reversible damage in periodontal tissues. *Capnocytophaga* was among the 39 ‘core’ genera shared between the groups, present in at least 90% of samples, and *C. gingivalis* and *C. ochracea* were among the ‘core’ species found in cases [54]. Acharya et al. [55] examined salivary microbiomes of 20 treated periodontitis patients and 15 periodontally healthy individuals, using a species-level resolution approach in their search for potential species able to separate individuals with well-maintained periodontal treatment from healthy individuals with similar gingival inflammation levels. *C. leadbetteri* was among the four species that were found to be significantly more abundant in the treated periodontitis group [55].

It is plausible that the increased pathogenicity within dental biofilms occurs due to microbial shifts with decreased or increased abundances of commensal bacteria. A recent study used a combination of high-throughput 16S rRNA gene amplicon sequencing and species-specific PCR primers targeting 10 different species of potential periodontal pathogens, including *C. ochracea* and *C. sputigena*, to investigate how various species and genera affected the colonization of ‘red complex’ species within dental plaque collected from older adults [56]. Detection rates of *C. ochracea* and *C. sputigena* were significantly higher in individuals positive for *Tannerella forsythia* and *Porphyromonas gingivalis*, respectively, than in those negative for these periodontal pathogens. It may be that individuals colonized with detectable levels of red complex species in dental biofilms—potentially supported by less pathogenic species like *Capnocytophaga—*are susceptible to harmful consequences for their oral and systemic health.

Analyzing vast genome-wide association study (GWAS) datasets from 455 documented tongue dorsum microbiomes and 540 documented saliva microbiomes in an East Asian cohort, He et al. [57] aimed to identify the causal relationship between the oral microbiome and five types of respiratory infections, including tonsillitis, chronic sinusitis, bronchiectasis, and pneumonia. Five genera, *Prevotella*, *Streptococcus*, *Fusobacterium*, *Pauljensenia*, and *Capnocytophaga* proved to play crucial but varied roles, either dampening or intensifying the progression of infection. Of these, *Capnocytophaga* (*C. leadbetteri* and *C. ochracea*) was found to inhibit bronchitis and pneumonia [57]. In another study focusing on the tonsillar crypt microbiota, the bacterial diversity within the crypts of the palatine tonsils was examined using 16S rRNA gene amplicon pyrosequencing in children and adults with recurrent tonsillitis, children with tonsillar hyperplasia, and healthy adults with surgery other than tonsillectomy (n = five per group) [58]. *Capnocytophaga* was one of the most prevalent genera with four species and non-distinguishable groups of species. Of the 33 species exceeding an average mean abundance of >0.5%, *C. leadbetteri* was recovered from 13/20 tonsillar samples, with two to four samples being positive in each group.

Of special note, in a recent comprehensive analysis of how spaceflight (on the SpaceX Inspiration4 mission, Dragon capsule) may affect the human microbiome [59], researchers noted a massive transient decrease in the relative metatranscriptomic activities of *C. granulosa* during spaceflight, the largest decrease out of all oral taxa studied. The authors proposed that this may reflect changes in microbial nutrient requirements or host dietary changes occurring during spaceflight.

## 3. Coaggregation and Synergistic Biofilm-Forming Activities with Different Oral Bacterial Species

Pioneering in vitro studies revealed that *C. ochracea*, *C. sputigena*, and *C. gingivalis* cells exhibited a variety of inter-generic cell coaggregation activities with gram-positive species that are common components of oral biofilms, including certain *Streptococcus*, *Actinomyces*, and *Rothia* species [60]. Broadly speaking, *C. ochracea* exhibited the greatest range of inter-species binding capacities [61]. Interactions were putatively mediated by high-molecular-weight, heat-sensitive ‘lectin-like’ surface proteins (also present on *C. gingivalis* cells), which exhibited differing degrees of inhibition with ca. 5–100 mM concentrations of sugars, e.g., N-acetylneuraminic acid, N-acetylglucosamine, L-rhamnose, and L-fucose, in a species-specific manner [62]. It was proposed that the binding of facultative anaerobes such as *C. ochracea* to ‘pioneer’ species in nascent biofilms may drive further oral biofilm maturation, notably via binding to the key ‘bridging’ species *Fusobacterium nucleatum* [63].

The coaggregation between *C. ochracea* and *F. nucleatum* cells could be inhibited by the addition of EDTA or lysine, but not by (amino-)sugars or arginine, and occurred via a heat- and proteinase K-sensitive component on *F. nucleatum* cells. Later studies revealed that *C. ochracea* formed a synergistic biofilm with *F. nucleatum*, and a secreted *C. ochracea* product (putatively the quorum sensing autoinducer AI-2) stimulated *F. nucleatum* biofilm formation [64]. More recently, *C. ochracea* was found to co-aggregate with the periodontal pathogen and oncobacterium, *Parvimonas micra*, and stimulate its growth in an apparently indirect manner, e.g., through a secreted product, possibly AI-2 [65].

*Capnocytophaga* cells are highly motile on surfaces and often exhibit a highly distinctive pattern of swirling and collective swarming behaviors [1,66,67]. They do not utilize flagellae or pili to ‘swim’ or ‘twitch’ like many bacterial species, but locomote via gliding. The complex, multi-component cell surface gliding machinery is powered by the type IX secretion system (T9SS), a rotary motor that secretes adhesins onto the outer membrane surface in a helical manner [68]. During this gliding process, other bacteria and even bacteriophage may ‘hitchhike’ on the long *Capnocytophaga* cells [67,69]. For example, *C. gingivalis* could translocate seven different non-motile species of oral bacteria, including *Actinomyces* sp. HMT-169, *Veillonella parvula*, *Streptococcus sanguinis*, *P. micra*, *Porphyromonas endodontalis*, *Prevotella oris*, *and F. nucleatum* [67]. This is thought to be a key process by which *Capnocytophaga* may drive dynamic biofilm growth, maturation, and spatial ecology.

## 4. Biogeography and Pangenome of *Capnocytophaga* Species in Human Oral Niches

### 4.1. Biogeography

*Capnocytophaga* is among the top 10 most common genera typically present in dental plaque [70]. Furthermore, pioneering RNA-sequencing studies revealed that *Capnocytophaga* species are highly transcriptionally active within (supragingival) dental plaque biofilms, in many cases constituting ca. 10% of all bacterial transcripts [71]. Thus, *Capnocytophaga* plays major roles in affecting the compositions and collective activities of oral biofilms. In their groundbreaking biogeographical analysis, Mark Welch et al. [72] used combinatorial labeling and spectral imaging fluorescence in situ hybridization (CLASI-FISH) to study the 3D architectures and taxonomic compositions (i.e., spatial ecology) of oral biofilms, and found *Capnocytophaga* to be the most abundant taxon present in dental plaque, being considerably more prevalent in supragingival compared with subgingival plaque. Notably, *Capnocytophaga* taxa were regular components of ‘hedgehog’ structures, which are commonly observed in dental biofilms, especially within a wide, putatively oxygen-depleted band in the peripheral regions, just below the surface layer [72]. *Capnocytophaga* taxa were also reported to be abundant in the peripheral regions of filament-rich annular (ring-like) components around ‘corncob’ structures, alongside *Fusobacterium*, *Leptotrichia*, and *Streptococcus*, whose central stalks were formed by *Corynebacterium* cells. Whilst their CLASI-FISH approach did not enable species-level identification of the *Capnocytophaga* taxa present, their findings reconcile well with what is known about the ecological preferences of *Capnocytophaga* (e.g., CO_2_-enriched low-oxygen niches), as well as with results from previous cell-coaggregation and synergistic growth studies involving *C. ochracea* (and to a lesser extent, *C. sputigena* and *C. gingivalis*) as noted above [72].

### 4.2. Relationships Between the Pangenome and Biogeography

Recently, Giacomini et al. [26] assembled a high-quality set of *Capnocytophaga* reference genomes (*n* = 117) from available genomic and metagenomic sequence data. From this, a human *Capnocytophaga* pangenome of 13,954 gene clusters was identified, with 341 core genes (gene clusters present in all genomes) and 9493 accessory genes (gene clusters present in 2–116 genomes). They revealed that the species *C. sputigena*, *C. leadbetteri*, *C. granulosa*, and *C. gingivalis* each formed two different genomic groups, based on their respective genomic compositions. By mapping specific gene-sequence reads to metagenomic data from 10 different oral niches, they were able to survey the relative abundance of *Capnocytophaga* species and genomic groups present within these respective niches. The two different genomic groups of *C. sputigena*, *C. granulosa*, and *C. gingivalis* exhibited dichotomous behaviors, with one set preferentially occupying the preferred niche of supra- and subgingival plaque, whilst the other atypical set occupied the tongue dorsum. Further (KEGG-based) predicted metabolic analysis suggested that all *Capnocytophaga* taxa shared many commonalities, such as carbohydrate (notably glucose) metabolism, bicarbonate (CO_2_) utilization, and conserved cofactor and vitamin biosynthetic capabilities. However, certain outlier groups within specific species may possess notably different metabolic adaptations, e.g., differences in oxygen utilization, nitrate/nitrite metabolism, or (aromatic) amino acid biosynthesis capabilities. The authors proposed that a single *Capnocytophaga* species may encompass multiple distinct genomic compositions that adapt its physiology to life in different oral niches or environments [26].

In a study by Koohi-Moghadam et al. [73], metagenomics was used to analyze biosynthetic gene clusters (BGCs) encoded by bacterial taxa present in saliva and supra- and subgingival plaques from periodontitis patients and healthy controls. Several BGCs are associated with periodontal health and disease. Notably, they identified one BGC (k141_164411) predicted to synthesize a bioactive polyketide metabolite within *C. gingivalis* contigs that were uniquely obtained from supra- and subgingival plaque from seven individuals with periodontitis, but were completely absent from healthy controls. This highlights the power of using genomics and metagenomics for analyzing the ‘accessory genome’ of oral bacteria to identify putative novel factors underlying health or disease [73].

## 5. Clinical Relevance of *Capnocytophaga* in the Oral Cavity

### 5.1. Periodontal Disease

In individuals suffering from periodontitis, an infection-driven inflammatory disease affecting tooth-supporting tissues, the taxonomic compositions and collective biological activities of subgingival microbial communities differ from those in periodontally healthy individuals [74]. *Capnocytophaga* levels are typically associated with periodontal health [75,76]. However, the detailed picture is more intricate and variable, and the precise roles played by individual *Capnocytophaga* species within diverse oral communities remain somewhat controversial. On one hand, *Capnocytophaga* (especially *C. gingivalis* and *C. granulosa*) is one of the genera detected at high frequencies in subgingival sites of individuals with gingival inflammation [77,78] and thus considered to have a potential pathogenic role in periodontal disease progression [79,80] and in gingivitis and periodontitis in diabetic children and adults [39,81]. Also, *C. granulosa* and *C. haemolytica* have been found in subgingival plaque of 51% and 10% of 29 periodontitis patients examined, respectively [82]. It should also be noted that the two strains of the recently described *C. periodontitidis* species were isolated from deep periodontal pockets [13]. A study by Nibali et al. [83] examined the periodontal microbiome in patients with aggressive periodontitis following treatment. Whilst *C. granulosa* (HMT-325) levels and the total amount of all *Capnocytophaga* taxa present within periodontal sites were positively associated with persistent disease (over ca. seven years), *C. sputigena* (HMT-775) levels were positively associated with a long-term successful treatment outcome [83]. In studies by Duran-Pinedo et al. [84,85], metatranscriptomics was used to analyze changes in the composition and activity of stable and progressing periodontal sites following non-surgical treatment in individuals with grade II/III periodontitis. Amongst a complex set of findings, *Capnocytophaga* sp. HMT-864 and HMT-338 belonged to clusters that were exclusively associated with progressing periodontal sites. However, 12 different species and HMT-phylotypes of *Capnocytophaga* (including *C. gingivalis*, *C. granulosa*, *C. ochracea*, *C. sputigena*, and *C. leadbetteri*) were associated with clusters that were exclusively associated with stable sites. Taken together, the results revealed a complex and dynamic relationship between the composition and activities of the bacterial communities and the respective physiological responses within periodontal niches, where pro-disease and pro-health periodontal microbiome communities may have many different structures.

A recent study using metagenomics aimed to investigate potential differences in subgingival microbial diversity, composition, interaction networks, and functional potential between three periodontal states, severe periodontitis (stage III) with moderate risk of progression (grade B) and high risk of progression (grade C) in comparison with periodontal health [86]. Interesting differences were seen between grades B and C, the latter having greater microbial complexity and a marked enrichment of *C. granulosa* and *Capnocytophaga* sp. CM59 (HMT-471), indicating a potential association with more rapid progression. In a complex microbial co-occurrence network in grade C periodontitis individuals, *C. ochracea* appeared as a hub species with key members of Socransky’s red complex but had a negative correlation with several pathogenic species. *C. leadbetteri*, *C. sputigena*, *C. granulosa*, and *Capnocytophaga* sp. CM59 (HMT-471) were among the top 50 ‘species’ in subgingival plaque, but without significant relationships to clinical indicators (in this study: bleeding on probing, pocket depth, and clinical attachment level) [86]. Furthermore, in another recent study, the abundance of *Capnocytophaga* exhibited a significant decreasing trend when moving from health to severe (stage III) periodontitis with increasing pocket depths of 0–3 mm, 4–5 mm, and 6–9 mm [87]. *C. gingivalis*, *C. granulosa*, and *C. sputigena* appeared in a co-occurrence network of species with a relative abundance of >1% in the healthy and pocket depth of 0–3 mm groups, and in the latter group, *C. gingivalis* and *C. sputigena* had a strong positive correlation with *Neisseria elongata* and each other. Being less abundant, *C. leadbetteri* and *C. endodontalis* (*Capnocytophaga* genospecies AHN8471/*C. periodontitidis*) appeared in the 4–5 mm group, having a weak correlation with each other [87].

In the systematic review and meta-analysis of Antezack et al. [88], the prevalence of potentially pathogenic microorganisms in subgingival plaque and/or saliva was compared between individuals with and without periodontitis. In the forest plot of the effect sizes for the association between microorganisms and periodontitis, the odds ratios (95% confidence intervals) for *C. gingivalis*, *C. ochracea*, and *C. sputigena* were 1.02 (0.65–1.61), 1.10 (0.74–1.63), and 1.35 (0.69–2.63), respectively. In other words, no statistically significant associations between these *Capnocytophaga* species and periodontitis were present [88]. Recently, Xu et al. [89], by analyzing 16S rRNA gene sequencing data, focused on identifying subgingival microbial taxa differences that may serve as potential drivers of increasing severity of periodontitis. *Capnocytophaga* and *Paludibacter* were identified as protective genera, significantly enriched in the healthy group. Interestingly, *Capnocytophaga* was among the genera, and, at the species level, *C. leadbetteri* demonstrated a decreasing correlation with periodontitis progression. In terms of microbial community re-organization, there was a gradual loss of health-associated taxa and a sequential emergence of disease-associated pathogens. In addition, microbial co-occurrence networks were less stable and resilient in advanced periodontitis and less likely to return to their original equilibrium state, compared to mild and moderate disease stages or health [89].

### 5.2. Periimplantitis

Similar to periodontitis, periimplantitis is a common submucosal biofilm-associated disease that affects the tissues surrounding dental implants. In the peri-implant microbiome, shifts from health to disease include an increase in diversity and a gradual depletion of commensals [90]. With the aim of clarifying bacterial interactions, community structure, and microbial stability in dysbiotic biofilms, supra- and subgingival plaque samples were collected from teeth or implants of 34 individuals with different health conditions and examined using metagenomic sequencing [91]. *C. granulosa* and *C. sputigena* were among the hub species, playing central roles in different co-occurrence networks: *C. granulosa* in diseased subgingival communities and *C. sputigena* in healthy subgingival and diseased sub- and supragingival communities. *C. sputigena* had several negative correlations in health, but during inflammation, the correlations weakened and were lost. In diseased communities, *C. gingivalis* became a concentrated node of negative correlation. Notably, with increased inflammation, subgingival bacterial networks around teeth and implants became less connected and less competitive, whereas in supragingival communities, the networks shifted in the opposite direction [91].

Recently, the submucosal microbiome around implants was examined using full-length 16S rRNA gene amplicon sequencing and metatranscriptomics to identify bacterial taxa and their activities as potential driving factors for severe tissue destruction [92]. Similar to the situation in periodontitis, *Capnocytophaga* was among the genera having a negative correlation with increased disease severity, and significant associations between *C. gingivalis*, *C. sputigena*, and *C. leadbetteri* with shallower pockets were observed. However, in another study using full-length 16S rRNA sequencing [93], higher abundances of these *Capnocytophaga* species were found in peri-implant pocket depths of 5–7 mm, indicating early stages of periimplantitis.

### 5.3. Caries and Endodontic Infections

A recent systematic review presented data on the microbial composition and functional profile of dental plaque and/or saliva samples collected from caries-free and caries-affected individuals, based on 54 studies with approximately 3500 individuals [94]. Twelve bacterial genera, among them *Capnocytophaga*, were frequently reported as being more abundant in caries-active individuals. In root caries, increased levels of *C. granulosa* have been observed on carious teeth compared to non-carious teeth [95], indicating that *C. granulosa* has a pathogenic role. However, this finding is hard to reconcile with its health-associated status in supragingival plaque [96].

A large variety of bacterial species have been detected within primary endodontic infections without or with a sinus tract [97]. Specific differences in the bacterial composition existed between cases with or without a sinus tract; for instance, *C. sputigena* and *C. gingivalis* were detected in significantly higher counts in the absence of a sinus tract [97]. It should also be noted that the single strain of the novel species *C. endodontalis* was isolated from a human refractory periapical abscess [12].

Early microbial complex species connected to endodontic-periodontal lesions in the same tooth were the focus of a systematic review presenting geographically heterogeneous data coming from only four selected articles with a total of 116 teeth [98]. Prevalence rates of two *Capnocytophaga* species were available from two studies: for *C. granulosa,* 10% in samples taken from root canals and 35% from periodontal pockets, while corresponding rates for *C. sputigena* varied between 4–70% and 0–30%. As such, the evidence is obscure due to the limited data available.

### 5.4. Mucosal Infections

In immunocompromised patients, *Capnocytophaga* taxa have been found in oral and gingival mucosal lesions. Oral mucositis can appear as a specific chemotherapy-induced infection with mucosal ulceration, bleeding, and severe pain. This has been linked to distinct microbiome profiles within pediatric cancer patients, where the predominant phylum is Bacteroidota, and *Capnocytophaga* at the genus level, and the species *C. sputigena* may be present with significantly increased abundances [99,100,101]. According to a case report, a biopsy taken from gingival hyperplasia of a neutropenic woman demonstrated invasion of filamentous bacteria, identified as *C. sputigena*, within the gingival tissue [102]. As human oral-associated *Capnocytophaga* spp. most commonly originate from dental biofilms; one major route of entry to the bloodstream is through inflamed periodontal tissues [103,104]. Another route of translocation of oral bacteria can occur via (micro-)aspiration to the lower respiratory tract. For instance, Tan et al. [105] collected paired subgingival plaque and tracheal aspirate samples from 53 chronic obstructive pulmonary disease (COPD) patients with severe acute exacerbations. The presence of *C. sputigena* at equal levels in both sample types indicates its potential contribution to this severe condition. These observations underline the importance of proper oral hygiene and preventive measures for maintaining good oral health, especially in immunocompromised individuals [105].

### 5.5. Nitrate Reduction and Taste Perception

*C. gingivalis*, *C. ochracea*, and *C. sputigena* have all been shown to be active nitrate reducers and nitrite producers in dental plaque in adults and children [106]. The conversion of nitrate to nitrite by oral bacteria may play a significant role in helping protect against diabetes and hypertension [107]. A recent clinical study investigated changes in the oral microbiome and oral metabolome relating to taste perception in response to nitrate supplementation. Uniquely, levels of *C. gingivalis* were significantly increased in individuals whose taste perception did not change in response to nitrate supplementation [108]. The underlying mechanisms behind this interesting correlation remain to be elucidated.

## 6. Species-Level Identification of Human *Capnocytophaga* Recoveries from Clinical Samples

Bacterial identification methods and taxonomic classifications change and evolve over time. Consequently, care and caution should be employed when interpreting results from older studies or from studies that do not accurately report the experimental methodologies used for bacterial identification. Broadly speaking, there is relatively little experimental (clinical and laboratory-based) data on human *Capnocytophaga* species, compared with many other species of oral bacteria. Most pertinently, we lack a holistic understanding of their pathogenic potential and antibiotic susceptibility patterns, factors that may differ dramatically between species or within specific strain lineages.

Attempts to identify *Capnocytophaga* organisms to the species level often remain unreliable when identification relies on conventional phenotypic and biochemical tests, including colony morphology, Gram staining, yellow/orange pigmentation, gliding motility, CO2-requiring growth, sugar fermentation, and enzymatic activities [1]. In particular, the slow, fastidious growth requirements, along with phenotypic and biochemical similarities among *Capnocytophaga* species, limit the accuracy of species-level identification [15]. Before the introduction of molecular methods, culture-based identification, which required laborious testing procedures, could even fail to separate bacteria similar to *Capnocytophaga* [109]. According to the Centers for Disease Control and Prevention [110], blood samples are usually used to identify the bacteria in culture, but identification can be difficult because automated blood culture systems may miss *Capnocytophaga* due to insufficient incubation time. In other words, challenges in detection and identification most likely lead to an underestimation of the true prevalence of each *Capnocytophaga* species as a causative agent of infections. These limitations necessitated the introduction of more advanced molecular and proteomic technologies to improve species-level identification of *Capnocytophaga*.

Before the adoption of ‘next-generation’ DNA sequencing technologies, approaches such as PCR-restriction fragment length polymorphism combined with Sanger sequencing of near-full-length 16S ribosomal RNA (rRNA) amplicons were a powerful and reliable approach for the species-level identification of *Capnocytophaga* strains or taxa present in biological samples [33,111]. However, the 16S rRNA amplicon sequencing-based approach cannot give any information about the genetic/genomic factors that may be responsible for any biologically important or atypical phenotypes, e.g., surface antigens or antibiotic resistance profiles. It is also highly dependent on a large, diverse, and clinically representative set of reference strains and 16S rRNA gene sequences. Nowadays, the use of long-read sequencing platforms for the rapid and accurate determination of complete genome sequences for clinical isolates, e.g., using the Pacific Biosciences (PacBio) or Oxford Nanopore Technologies (ONT) platforms, is highly desirable to enable accurate taxonomic identification along with the identification of putative phages, mobile genetic elements, or antibiotic resistance genes [112].

Matrix-assisted laser desorption/ionization time-of-flight mass spectrometry (MALDI-TOF MS) is a molecular identification technique introduced in the 1990s, which is particularly useful for bacterial identification based on protein spectral fingerprints [113]. Concerning *Capnocytophaga*, the initial focus was on animal species, especially *C. canimorsus* [114], but later, it was extended to human *Capnocytophaga* species [115,116,117,118,119]. In hospital laboratories, MALDI–TOF MS is regarded as a rapid and cost-effective diagnostic method for microbes present in clinical samples [120]. It also reliably identifies *Capnocytophaga* to the genus level with 100% accuracy; however, its performance at the species level is variable. As shown by Algahawi et al. [116], the accuracy and speed of species-level identification of human oral-associated *Capnocytophaga* are strongly influenced by the representation of strains in the MALDI-TOF MS database, where an adequate number of representative *Capnocytophaga* strains is needed to assure the correct identification at both the genus and species levels without customizing protocols. Strains that are underrepresented in the MALDI TOF database or are closely related require additional preparation methods, such as formic acid extraction, and the database should be enriched with these species [116]. Thus, updating and expanding the MALDI-TOF MS reference database will further improve the species-level identification accuracy of *Capnocytophaga* in clinical microbiology laboratories.

## 7. Human Oral-Associated *Capnocytophaga* Species in Non-Oral Infections

Clinically, the *Capnocytophaga*-associated disease can progress rapidly from mild, localized infection to systemic infection, sepsis, and even death [110].

### 7.1. Bloodstream Infections

Outside the oral cavity, most human oral-associated *Capnocytophaga* species have been isolated from blood. Bloodstream infections form the major infectious type with *Capnocytophaga* involvement, especially in individuals with weakened immune systems [121,122]. Recently, Chesdachai et al. [122] performed a retrospective review of human- and animal-associated *Capnocytophaga* infections, covering a 10-year period at three main hospitals of the Mayo Clinic. A total of 110 adult patients with culture-positive *Capnocytophaga* findings were recognized, and of them, 82 were connected to a clinically relevant infection. Among the 62 *Capnocytophaga* isolates identified to the species level, *C. sputigena* was most frequent (45%), and, in descending order, was followed by zoonosis-associated *C. canimorsus* (26%), *C. ochracea* (16%), and *C. gingivalis* (8%). In addition, one *C. granulosa*, one *C. leadbetteri*, and one zoonosis-associated *C. cynodegmi* were found. Nearly all bacteremia cases were monomicrobial, where *C. sputigena* was the most common finding, followed by *C. canimorsus*, but also *C. ochracea* and *C. gingivalis* were occasionally detected. Most non-bacteremia cases were polymicrobial in nature, with human oral-associated *Capnocytophaga* forming part of their infectious etiology. Notably, infections with human-associated *Capnocytophaga* have been proven to have a higher rate of overall six-month mortality compared to that of *C. canimorsus* [122]. Recently, an unusual septicemia case caused by *C. ochracea* after a dog bite was described [123]. According to the authors, this was the first report of bacteremia where human oral-associated *Capnocytophaga* (identified as *C. ochracea* using MALDI-TOF) was transmitted from an animal. It was speculated that transmission can occur in both directions due to close contact between humans and their pets.

In a recent review of 31 infective endocarditis cases involving *Capnocytophaga* species [124], only five case reports were connected to human oral-associated species; three with *C. ochracea* and one with *C. haemolytica* (identifications based on phenotypic tests). There is one further bacteremia case potentially connected to endocarditis caused by *Capnocytophaga* genospecies AHN8471 [125] available in the literature. As described above, the uncertain (overlapping) taxonomic positions of *Capnocytophaga* genospecies AHN8471, *C. endodontalis*, and *C. periodontitidis* should be well noted.

An especially frequent recovery from bloodstream infections seems to be *C. sputigena*. In both adult and pediatric patients with hematologic malignancies, there are several bacteremia cases involving *C. sputigena* [100,117,126,127,128,129,130,131,132,133]. In a catheter-related bloodstream infection caused by *C. sputigena*, the isolate from the patient was tested for its biofilm formation and compared with the laboratory reference strain; this clinical isolate proved to have significantly increased biofilm formation capability, which was interpreted as a pathogenic mechanism [134]. An interesting, historic sepsis case of Saint-Louis, King of France (1270 AD) was recently reported by Charlier et al. [126]; the king was assumed to suffer from scurvy and inflammatory jaw disease with the potential involvement of oral commensals. Based on careful molecular analyses of a sample from the visceral relics, *C. sputigena* was identified as the main pathogenic species with a uniquely high proportion (11%) of all microbial sequences. Thus, it was suggested that *C. sputigena* was responsible for the fatal bacteremia, leading to the king’s death [126].

### 7.2. Other Types of Non-Oral Infections

Sporadic case reports have been presented on infections with the involvement of human oral-associated *Capnocytophaga* species affecting the eye, central nervous system, and the upper and lower respiratory tracts, but also those affecting the female genital tract and the fetus during pregnancy. In addition to blood, *C. sputigena* seems to be the most common also in these other infectious sites outside the oral cavity. Out of 49 clinical β-lactamase-positive *Capnocytophaga* strains from patients treated in a Japanese university hospital, 31 identified as *C. sputigena* were isolated from 29 respiratory samples, one from a wound specimen, and one from blood [135]. Reports on non-oral infections vary from orbital cellulitis and adjacent subperiosteal abscess [136], sinusitis with orbital subperiosteal and intracranial abscesses [137], tonsillitis [128], community-acquired pneumonia [138], pneumonia [132], pleural empyema [139,140], and lung abscess [141,142] to soft tissue or osteo-articular infections; for instance, post-operative intra-abdominal abscess [143], primary iliopsoas abscess [144] as well as post-arthroscopic knee joint infection connected to *C. sputigena* have been reported [145].

In a pre-term birth case series report by Lopez et al. [146], *C. sputigena* was detected in two out of five cases. In case 1, the pregnancy was uncomplicated until the premature rupture of membranes occurred at 25 weeks of gestation, followed by preterm birth with low birth weight at 26 weeks of gestation. Chorioamnionitis was clinically suspected and confirmed by histological examination of the placenta. The infant was treated for respiratory distress syndrome, and *C. sputigena* was isolated from the tracheal aspirate and gastric fluid cultures, whereas the blood culture was negative. In case 2, an infant with low birth weight was delivered at 28 weeks of gestation. *Trichomonas vaginalis* was recovered in vaginal cultures of the mother. Chorioamnionitis was clinically suspected and confirmed by histological examination of the placenta. Blood culture and gastric aspirate samples revealed the presence of *C. sputigena* [146]. Similar cases related to chorioamnionitis and *C. sputigena* involvement alone [147] or together with *C. leadbetteri* [148] have been observed as well. Also, *C. ochracea* was linked with preterm birth and neonatal septicemia, as it was detected in the blood culture of a premature neonate [149]. In cases affecting the urogenital tract, insufficient oral hygiene and poor oral health status may be regarded as background factors resulting in the transfer of oral bacteria via hematogenous routes or oral sex to the female genital tract, where *Capnocytophaga* organisms are able to cause infection [146,147,150].

Besides *C. sputigena*, other traditional *Capnocytophaga* species, *C. ochracea* and *C. gingivalis*, are sporadic findings in a variety of non-oral infections. One explanation might be the challenges connected to their identification, as shown in a leukemia patient with pleural effusion and empyema, where anaerobic gram-negative bacilli were isolated from pleural fluid after three days of incubation, but it was not until 15 days of hospitalization that *C. ochracea* was identified by the reference laboratory [151]. Notably, as shown in a recent case report on *C. gingivalis*, bacteria with low-level pathogenicity can lead to a fatal outcome [152]. It was speculated that co-infection with COVID-19 with massive lung destruction was associated with *C. gingivalis* colonization of the lungs, thus leading to fulminant sepsis. Also, the recently reported first-ever case of central nervous system infection caused by *C. ochracea*, where a surgical operation through the oral and nasal passages was suspected to have interfered as a concomitant factor, is noteworthy [153]. In the study, a variety of methods, including metagenomics, next-generation sequencing, MALDI-TOF MS, and 16S rDNA sequencing, were used to identify the isolate from the cerebrospinal fluid of a meningioma patient. Indeed, next-generation sequencing (NGS) is a valuable method for identifying fastidious bacteria in polymicrobial infections, such as brain abscesses of odontogenic origin, where *Capnocytophaga* can also be found [154]. In this case, the growth from pus specimens was identified by MALDI-TOF MS as *C. ochracea* and another species, *Arachnia propionica.* The post-mortem pus sample was further analyzed using 16S deep sequencing, recognizing a wide range of oral anaerobic species, including *Capnocytophaga* sp. HMT-323, in this polymicrobial brain abscess [154]. In a diabetic patient with poor oral hygiene, bacteremia followed laparoscopic gastrectomy for gastric cancer, and the bacterial isolate from blood was then identified by MALDI-TOF and confirmed by 16S rRNA gene sequencing as *C. ochracea* [155]. This diabetic patient was estimated to have an increased risk of *Capnocytophaga* infection due to poor glycemic control and chronic inflammatory burden in periodontal tissues.

Infectious cases caused by *Capnocytophaga* are recognized in both immunocompetent and immunocompromised individuals [121,156]. A literature review collected data on 43 systemic infections associated with human oral *Capnocytophaga* species, published in English up to February 2016 [156]. Altogether, 25 *C. ochracea*, 12 *C. sputigena*, four *C. gingivalis*, one *C. leadbetteri*, and one *C. haemolytica* case were described; however, the precise species-level identification remains uncertain when based on phenotypic testing or when the methodology was not described in detail. For instance, out of the 25 publications reporting the isolation of ‘*C. ochracea* from non-oral infections, 12 came from studies published in the 1980s, and 6 were from the 1990s, which predates the description of several species closely related to *C. ochracea*. An interesting observation was that 7 of the 43 systemic infectious cases were connected to oral conditions [156].

The first septic arthritis case caused by *C. gingivalis* was reported in an immunocompetent young child with positive cultures from subtalar joint fluids at two separate times and identified at a reference laboratory, leading to a considerable delay in diagnosis and treatment [157]. A potential mechanism was suspected to occur via the hematogenous route from profuse gingival bleeding due to oral trauma to the subtalar joint. One *C. granulosa* case in an immunocompetent teenage boy, consisting of an abscess in the lumbosacral area after trauma, was reported based on extensive phenotypic tests [158]. The first report of a Bartholin’s gland abscess caused by *C. ochracea* was presented in an immunocompetent woman [159].

Besides the challenging diagnosis of slow-growing *Capnocytophaga* organisms to the species level, antimicrobial susceptibility testing can be a difficult task for clinical microbiology laboratories that may lead to delays in the initiation of effective antimicrobial therapy. As suggested by Ehrmann et al. [160], human oral-associated *Capnocytophaga* species form a significant β-lactamase resistance gene reservoir in the oral microbiome; the most common β-lactamase producers were *C. sputigena* and *C. ochracea*, but β-lactamase production was also detected among *C. gingivalis*, *C. granulosa*, and *C. leadbetteri* isolates. A few macrolide-lincosamide-streptogramin-resistant *C. sputigena* and one *C. ochracea* were observed [160]. Moreover, among the 10-year hospital-based data with 82 clinically relevant infections caused by *Capnocytophaga*, 30% of strains produced β-lactamases [122]. A multidrug-resistant *C. ochracea* strain was isolated from the blood of a boy with leukemia and later characterized as positive for a plasmid-encoded TEM-17 extended-spectrum β-lactamase [161]. In an acute exacerbation of chronic obstructive pulmonary disease, a multidrug-resistant *C. gingivalis* strain was found in an elderly man with urgent acute respiratory distress [156]. In a patient with severe neutropenia, *C. gingivalis* isolated from blood proved to produce the CSP-1 extended-spectrum-β-lactamase [162]. Taken together, this underscores the importance of susceptibility testing for selecting effective antimicrobial therapy for human oral-associated *Capnocytophaga* species.

## 8. Concluding Remarks and Future Perspectives

Dental plaque is the primary site for human oral-associated *Capnocytophaga* species. The majority of human *Capnocytophaga* species generally play protective, health-promoting roles in dental plaque and other oral biofilm niches. However, there is a paucity of mechanistic molecular evidence demonstrating exactly how they do this. We speculate that a complex network of inter-species cell-binding activities and physiological interactions between *Capnocytophaga* (especially *C. ochracea*) and other commensals helps stabilize oral biofilm structure and community composition, underpinning oral biofilm resilience and eubiosis. However, a moderate amount of evidence suggests that *C. granulosa* and *C. gingivalis* may play notable (accessory) roles in oral dysbiosis and disease.

In subgingival biofilms, *Capnocytophaga* is significantly enriched in health but shows a decreasing pattern from health towards disease. An interesting comparison between the subgingival microbiomes in young adults with cryptogenic ischemic stroke and stroke-free controls revealed a significantly decreased abundance of *Capnocytophaga* in patients [163]. Notably, in the stroke group, individuals lacking symptoms at admission and those with favorable clinical outcomes had significantly enriched populations of *Capnocytophaga*. To date, however, relatively little is known about this oral genus and its potential link to this and other systemic conditions. Continued progress in the determination of complete genome sequences for diverse reference strains and clinical isolates of *Capnocytophaga*, combined with advances in the scale and depth of high-quality clinical molecular investigations, will give us fresh insight into the relevance of this understudied group of bacteria for oral and systemic health.

The number of immunocompromised individuals will continue to increase in future years, for instance, due to the development and implementation of new immunotherapies and targeted cellular therapies, thus increasing the risk of infections caused by low-level pathogens like *Capnocytophaga*. Based on recent reports in the current literature, using more advanced identification techniques in clinical microbiology laboratories, the most frequent *Capnocytophaga* species involved in human infections seems to be *C. sputigena*. In contrast, *C. haemolytica* is one of the least frequently detected human oral-associated *Capnocytophaga* species. There are still many controversies regarding the clinical relevance and roles of this oral genus; thus, greater emphasis should be given to *Capnocytophaga* in future studies.

## Data Availability

No new data were created or analyzed in this study. Data sharing is not applicable to this article.
